# Mechanical Debridement of Methicillin-Sensitive Staphylococcus aureus Endocarditis: A Novel Approach Using Percutaneous Aspiration

**DOI:** 10.7759/cureus.100022

**Published:** 2025-12-24

**Authors:** Muhammad Khakwani, Ahmad Jalil, Zahra Hassan, Maria Khakwani, Qandeel Anwar, Fatima Rajab, Vishal Sachdev

**Affiliations:** 1 Internal Medicine, North Mississippi Medical Centre, Tupelo, USA; 2 Internal Medicine, Baptist Memorial Hospital-North Mississippi, Oxford, USA; 3 Internal Medicine, Lahore Medical and Dental College, Lahore, PAK; 4 Internal Medicine, King Edward Medical University, Lahore, PAK; 5 Thoracic Surgery, North Mississippi Medical Centre, Tupelo, USA

**Keywords:** angiovac, cardiology, infective endocarditis, mssa bacteremia, percutaneous vegetation debulking, right-sided endocarditis

## Abstract

Infective endocarditis (IE) remains a serious and potentially fatal diagnosis with substantial morbidity, particularly when involving large right-sided vegetations or occurring in patients who are poor candidates for surgical intervention. Despite advancements in antimicrobial therapy and diagnostic imaging, management of these complex cases continues to pose significant clinical challenges. The AngioVac aspiration system (AngioDynamics, Inc., Latham, New York, United States) has emerged as a minimally invasive option capable of percutaneously removing intracardiac vegetations, thrombi, or infected material while maintaining hemodynamic stability through extracorporeal filtration and reinfusion. By reducing overall bacterial burden and mitigating the inoculum effect, this technique can enhance antibiotic efficacy, decrease the risk of septic embolization, and serve as a valuable bridge or alternative to surgery. We describe a case of severe right-sided IE complicated by persistent methicillin-sensitive *Staphylococcus aureus* (MSSA) bacteremia, septic pulmonary emboli, and multivalvular involvement in a young patient with significant clinical deterioration despite broad-spectrum antimicrobial therapy. Owing to prohibitive surgical risk, percutaneous vegetation debulking with the AngioVac system was pursued under transesophageal echocardiographic guidance. The procedure achieved a substantial reduction of vegetation size with immediate clinical and microbiologic improvement. Following AngioVac intervention, the patient demonstrated clearance of bacteremia, stabilization of respiratory status, and eventual recovery with continued targeted antimicrobial therapy. This case highlights the expanding role of AngioVac as an important therapeutic adjunct in select patients with right-sided IE, especially when conventional surgical management is not feasible. Continued reporting of such cases will help refine patient selection, procedural indications, and long-term outcomes for this evolving treatment modality.

## Introduction

Infective endocarditis (IE) is a fatal condition affecting many structures within the heart, including both native and prosthetic heart valves, endocardium, and implanted cardiac devices. The mortality rate of IE is up to 30% within the first year of diagnosis, making it a significant clinical challenge. Despite advancements in diagnostic and therapeutic techniques, the long-term prognosis for patients with IE has only slightly improved over the past several years. This highlights the complexity of managing IE and underscores the need for continued research into more effective treatment strategies [1].

The diagnosis of IE is made using a combination of clinical evaluation, imaging studies, and laboratory findings. The Duke criteria, particularly the major criteria, serve as the cornerstone for establishing an IE diagnosis. The presence of typical microorganisms, most commonly *Staphylococcus* or *Streptococcus* species, confirmed through four positive blood cultures, and the detection of valvular vegetations on echocardiography are key components of this diagnostic framework. Echocardiographic findings that support the diagnosis include mobile masses attached to valve leaflets, abscess formation, and new valvular regurgitation [2].

AngioVac (AngioDynamics Inc., Latham, New York, United States) is a treatment modality for IE that works by suctioning out vegetations and infected material from cardiac structures, including valves and implanted cardiac devices [3,4]. We present a case of a young female patient with right-sided IE who was treated using this device and demonstrated significant clinical recovery.

## Case presentation

A 20-year-old woman with a history of intravenous (IV) drug use presented to the Emergency Department (ER) with fever, body aches, and sore throat. On initial evaluation, her vital signs were: blood pressure 134/81 mmHg, heart rate (HR) 150 beats per minute, respiratory rate (RR) 32 breaths per minute, temperature 103.1°F, and oxygen saturation 96%. Laboratory testing revealed a white blood cell (WBC) count of 15.4 × 10⁹/L, hemoglobin 12.3 g/dL, and platelet count 253 × 10⁹/L. A rapid streptococcal antigen test was positive. She received a dose of ceftriaxone and was discharged on amoxicillin.

The patient re-presented three days later, with worsening fever, chills, generalized body aches, diarrhea, and a rash on her lower extremities. On examination, she was febrile, tachycardic, and appeared acutely ill. Vital signs were: blood pressure 123/84 mmHg, HR 131 beats per minute, RR 36 breaths per minute, temperature 99.9°F, and oxygen saturation 94%. Skin examination showed scattered papular and pustular erythematous lesions on both lower extremities. Lung examination revealed bilateral rhonchi. Laboratory findings at this time are summarized in Table 1.

**Table 1:** Laboratory test values on re-admission WBC: white blood cell count; AST: aspartate aminotransferase; ALT: alanine aminotransferase; INR: international normalized ratio; LDH: lactate dehydrogenase

Parameter	Value	Reference Range
WBC	36.1 ×10⁹/L	4.5–11.0 ×10⁹/L
Hemoglobin	9.6 g/dL	12.0–16.0 g/dL
Hematocrit	28.8%	36–46%
Platelets	52 ×10⁹/L	150–450 ×10⁹/L
Sodium	134 mmol/L	135–145 mmol/L
Potassium	3.3 mmol/L	3.5–5.0 mmol/L
Glucose	319 mg/dL	70–110 mg/dL
Creatinine	1.11 mg/dL	0.6–1.2 mg/dL
Albumin	2.6 g/dL	3.4–5.4 g/dL
Total Bilirubin	1.8 mg/dL	0.1–1.2 mg/dL
Alkaline Phosphatase	196 IU/L	44–147 IU/L
AST	51 IU/L	10–40 IU/L
ALT	27 IU/L	7–56 IU/L
INR	1.7	0.8–1.2
LDH	606 U/L	140–280 U/L
Lactic Acid	4.1 mmol/L (↓ to 2.2)	0.5–2.2 mmol/L
D-dimer	>20.00 µg/mL	<0.5 µg/mL

The complete blood count demonstrated leukocytosis with neutrophilic predominance and microcytic anemia. No schistocytes or platelet clumping were observed. Coagulation studies were not consistent with disseminated intravascular coagulation (DIC). A MedTox urine drug screen was positive for cannabinoids.

Blood cultures grew methicillin-sensitive *Staphylococcus aureus* (MSSA). Urine cultures were positive for *Klebsiella pneumoniae* and *S. aureus*. She was started on intravenous fluids, vancomycin, and azithromycin. Chest radiography revealed diffuse bilateral patchy infiltrates. Computed tomography angiography (CTA) of the chest ruled out pulmonary embolism but showed numerous cavitary pulmonary nodules consistent with septic emboli. Electrocardiography demonstrated sinus tachycardia with a HR of 131 beats per minute. Transthoracic echocardiography (TTE) revealed a preserved left ventricular ejection fraction (LVEF) of 65% and two distinct vegetations: one on the tricuspid valve measuring 1.9 × 1.4 cm and another in the right ventricular (RV) apex measuring 3.4 cm in length.

Despite broad-spectrum antimicrobial therapy including vancomycin and nafcillin, the patient remained febrile with persistent bacteremia and progressive clinical deterioration. Two day after her second presentation, she was intubated for respiratory distress and hypotension requiring vasopressor support. Repeat blood cultures continued to grow MSSA. Clindamycin was added due to concern for toxic shock syndrome, and ertapenem was initiated; however, no clinical improvement occurred. Clindamycin was subsequently discontinued, and nafcillin was replaced with cefazolin due to a better safety profile.

Repeat TTE demonstrated new vegetations on both the mitral and tricuspid valves and the absence of the previously noted finger-like mass in the RV apex.

**Figure 1:**
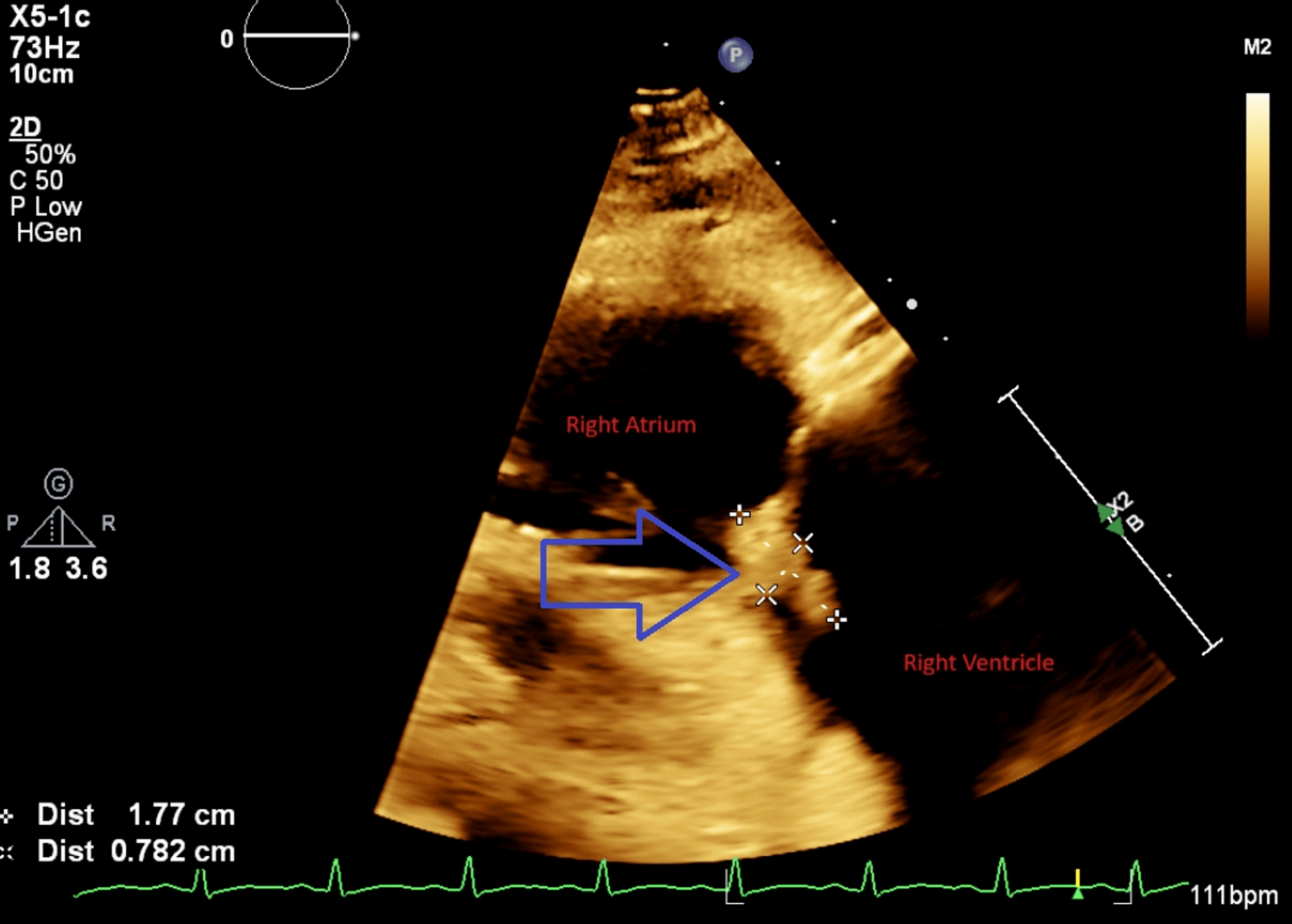
Transesophageal echocardiogram before AngioVac Blue arrow showing tricuspid vegetations (surrounded by white crosses)

**Figure 2:**
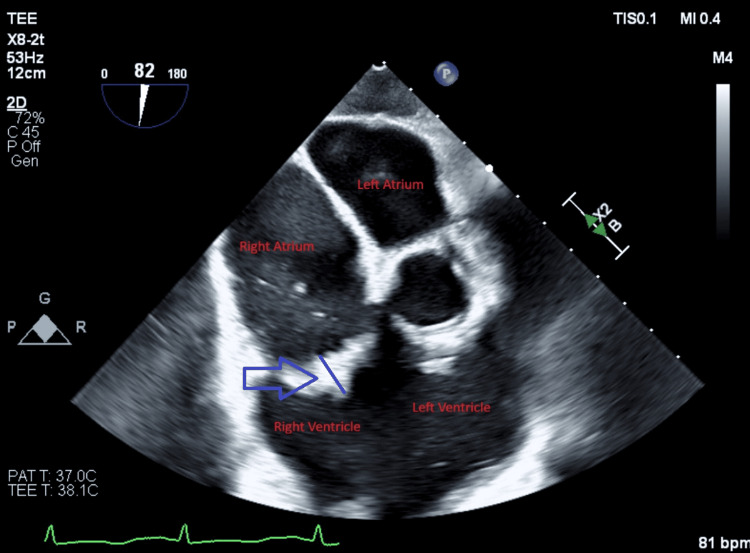
Transesophageal echocardiogram done after AngioVac Blue arrowhead showing decreased vegetations on tricuspid valve

Due to persistent bacteremia and large vegetations, cardiovascular surgery was consulted. The patient was deemed a poor surgical candidate, and percutaneous AngioVac debulking was planned. One week after the second presentation, under TEE guidance, the AngioVac system was used to remove vegetations from the tricuspid valve through right internal jugular and femoral venous access. Anaerobic cultures from aspirated material again grew MSSA. Figures 1, 2 show vegetations on the tricuspid valve before and after AngioVac evacuation, respectively.

Post procedure, the patient developed a diffuse skin rash and pleural effusions that required chest tube placement. Antimicrobial therapy was switched to daptomycin and linezolid. Blood cultures obtained after the AngioVac procedure remained negative. Patient remained hemodynamically stable. She was extubated five days after using the AngioVac system, and showed gradual clinical improvement.

The patient was discharged home a week later, with home health services. She received a seven-day course of levofloxacin and fluconazole, along with a six-week course of intravenous daptomycin and oral linezolid.

## Discussion

In 2014, the AngioVac system for the removal of unwanted intravascular materials, including thrombi, emboli, tumors, and septic vegetations from the right heart, was approved by the United States Food and Drug Administration. The system comprises a 22 French venous drainage cannula and a reinfusion cannula, both connected to an extracorporeal circuit with a pump head and bubble trap. When the bypass pump is activated, it generates suction that aspirates blood along with thrombotic or vegetative material into the cannula tip. The aspirated blood is then circulated through a filtration system before being reinfused into the patient through a second percutaneously placed venous cannula, typically inserted via the internal jugular or femoral vein. The large luminal diameter of the AngioVac cannula allows efficient removal of substantial thrombotic material while minimizing the risk of fragmentation and subsequent embolization [3-5].

High bacterial inoculum loads are associated with increased antibiotic resistance and reduced antimicrobial penetration, a phenomenon known as the inoculum effect [6]. By facilitating vegetation debulking, the AngioVac system helps reduce bacterial burden, thereby improving antibiotic effectiveness. This promotes faster resolution of sepsis and reduces hemodynamic compromise, a crucial factor influencing both operative and postoperative mortality in cardiac surgery [7]. According to Divekar et al., the AngioVac system provides a transcatheter option for critically ill patients with active right-sided IE, serving either as a bridge to more invasive surgical interventions or as a viable alternative in selected cases [3].

Several reports have documented the use of the AngioVac system in the management of IE. Patel et al. described that in cases of cardiac device-related IE (CDRIE) with large vegetations, using a percutaneous aspiration device before percutaneous lead extraction may reduce the risk of septic pulmonary embolism [8]. Jones et al. reported a case involving a 25-year-old woman with severe cardiomyopathy, an ejection fraction of 10%, and an implantable cardioverter-defibrillator who developed *Candida albicans* fungemia. Although European Society of Cardiology (ESC) guidelines recommend surgery for *Candida*-associated IE, she was deemed too high-risk and was treated with AngioVac. The system successfully aspirated the vegetation, her symptoms resolved, and with prolonged fluconazole therapy, her blood cultures remained negative for more than eight months [9].

George et al. analyzed the periprocedural outcomes of 33 patients with tricuspid valve endocarditis who underwent vegetation debulking using the AngioVac device. The mean vegetation size decreased from 2.1 ± 0.7 cm to 0.82 ± 0.5 cm after the procedure, representing an average reduction of 61%. All patients survived the procedure, and 90.9% remained free of reinfection and survived hospitalization, supporting both the safety and efficacy of the technique [10]. In another case series, Schaerf et al. evaluated 20 high-risk surgical patients with sepsis and CDRIE who had persistent vegetations despite optimal medical therapy. All patients underwent vegetation aspiration using the AngioVac system in conjunction with antimicrobial therapy, resulting in successful infection resolution [11].

Overall, available case reports and case series demonstrate considerable benefit of AngioVac-assisted debulking in the management of right-sided IE. As illustrated in our case, this technique offers meaningful clinical improvement in patients who are not suitable candidates for conventional surgical intervention. Having said that, there are a few complications also associated with this technique; the use of the AngioVac system necessitates large caliber venous access at two sites, most commonly involving a 26-French aspiration cannula and an 18-French reinfusion sheath, thereby predisposing patients to an increased risk of vascular and bleeding complications at access sites. Its deployment also requires close collaboration with an experienced perfusionist, as effective operation depends on familiarity with extracorporeal circuit setup and management and is associated with a procedural learning curve [12]. Moreover, AngioVac does not correct underlying valvular abnormalities and, in cases of IE, removal of bulky tricuspid valve vegetations may reveal or worsen tricuspid regurgitation [13].

A limitation of our case is the lack of long-term follow-up, which precludes assessment of durability, recurrence of infection, and late valvular outcomes following AngioVac-assisted vegetation debulking. Therefore, careful patient selection and a multidisciplinary heart team approach are essential to maximize clinical benefit while minimizing procedural risk when considering AngioVac-assisted debulking in right-sided IE.

## Conclusions

IE is a life-threatening condition, particularly when complicated by large right-sided vegetations, persistent bacteremia, and multivalvular involvement. In this case, despite appropriate antimicrobial therapy, the patient experienced continued clinical decline, demonstrating the limitations of medical management alone in certain high-risk situations. Surgical intervention is the standard approach for large vegetations; however, many patients are not suitable surgical candidates due to hemodynamic instability or prohibitive operative risk.

The use of the AngioVac aspiration system provided an essential alternative, allowing substantial reduction of vegetation burden, rapid clearance of MSSA bacteremia, and subsequent clinical recovery. This favorable response is consistent with growing evidence supporting AngioVac-assisted debulking as a viable therapeutic strategy for critically ill or surgically ineligible patients with right-sided IE. Continued reporting and future prospective studies are needed to refine patient selection, determine long-term outcomes, and define the optimal role of this evolving minimally invasive intervention.

## References

[1] Cahill TJ, Prendergast BD (2016). Infective endocarditis. Lancet.

[2] Habib G, Lancellotti P, Antunes MJ (2015). 2015 ESC guidelines for the management of infective endocarditis: the task force for the management of infective endocarditis of the European Society of Cardiology (ESC). Endorsed by: European Association for Cardio-Thoracic Surgery (EACTS), the European Association of Nuclear Medicine (EANM). Eur Heart J.

[3] Divekar AA, Scholz T, Fernandez JD (2013). Novel percutaneous transcatheter intervention for refractory active endocarditis as a bridge to surgery-angiovac aspiration system. Catheter Cardiovasc Interv.

[4] Abubakar H, Rashed A, Subahi A, Yassin AS, Shokr M, Elder M (2017). AngioVac system used for vegetation debulking in a patient with tricuspid valve endocarditis: a case report and review of the literature. Case Rep Cardiol.

[5] Moriarty JM, Rueda V, Liao M (2021). Endovascular removal of thrombus and right heart masses using the angiovac system: results of 234 patients from the prospective, multicenter registry of AngioVac procedures in detail (RAPID). J Vasc Interv Radiol.

[6] Rose WE, Leonard SN, Rossi KL, Kaatz GW, Rybak MJ (2009). Impact of inoculum size and heterogeneous vancomycin-intermediate Staphylococcus aureus (hVISA) on vancomycin activity and emergence of VISA in an in vitro pharmacodynamic model. Antimicrob Agents Chemother.

[7] Nakayama DK, O’Neill JA Jr, Wagner H, Cooper A, Dean RH (1986). Management of vascular complications of bacterial endocarditis. J Pediatr Surg.

[8] Patel N, Azemi T, Zaeem F, Underhill D, Gallagher R, Hagberg R, Sadiq I (2013). Vacuum assisted vegetation extraction for the management of large lead vegetations. J Card Surg.

[9] Jones BM, Wazni O, Rehm SJ, Shishehbor MH (2018). Fighting fungus with a laser and a hose: management of a giant Candida albicans implantable cardioverter-defibrillator lead vegetation with simultaneous AngioVac aspiration and laser sheath lead extraction. Catheter Cardiovasc Interv.

[10] George B, Voelkel A, Kotter J, Leventhal A, Gurley J (2017). A novel approach to percutaneous removal of large tricuspid valve vegetations using suction filtration and veno-venous bypass: a single center experience. Catheter Cardiovasc Interv.

[11] Schaerf RH, Najibi S, Conrad J (2016). Percutaneous vacuum-assisted thrombectomy device used for removal of large vegetations on infected pacemaker and defibrillator leads as an adjunct to lead extraction. J Atr Fibrillation.

[12] Jaber WA, Fong PP, Weisz G (2016). Acute pulmonary embolism: with an emphasis on an interventional approach. J Am Coll Cardiol.

[13] Baddour LM, Weimer MB, Wurcel AG (2022). Management of infective endocarditis in people who inject drugs: a scientific statement from the American Heart Association. Circulation.

